# Cohort Analysis of 67 Charcot-Marie-Tooth Italian Patients: Identification of New Mutations and Broadening of Phenotype Expression Produced by Rare Variants

**DOI:** 10.3389/fgene.2021.682050

**Published:** 2021-07-19

**Authors:** Rosangela Ferese, Rosa Campopiano, Simona Scala, Carmelo D’Alessio, Marianna Storto, Fabio Buttari, Diego Centonze, Giancarlo Logroscino, Chiara Zecca, Stefania Zampatti, Francesco Fornai, Vittoria Cianci, Elisabetta Manfroi, Emiliano Giardina, Mauro Magnani, Antonio Suppa, Giuseppe Novelli, Stefano Gambardella

**Affiliations:** ^1^IRCCS Neuromed, Pozzilli, Italy; ^2^Laboratory of Synaptic Immunopathology, Department of Systems Medicine, Tor Vergata University, Rome, Italy; ^3^Center for Neurodegenerative Diseases and the Aging Brain, Department of Clinical Research in Neurology, The University of Bari “Aldo Moro,” “Pia Fondazione Card G. Panico” Hospital Tricase, Lecce, Italy; ^4^Department of Basic Medicine Neuroscience and Sense Organs, University “Aldo Moro” Bari, Bari, Italy; ^5^Genomic Medicine Laboratory, IRCCS Fondazione Santa Lucia, Rome, Italy; ^6^Department of Translational Research and New Technologies in Medicine and Surgery, University of Pisa, Pisa, Italy; ^7^Regional Epilepsy Centre, Great Metropolitan Hospital Bianchi-Melacrino-Morelli, Reggio Calabria, Italy; ^8^Department of Neuroscience- Neurogenetics, Santa Maria Hospital, Terni, Italy; ^9^Department of Biomedicine and Prevention, University of Rome “Tor Vergata,” Rome, Italy; ^10^Department of Biomolecular Sciences, University of Urbino “Carlo Bo,” Urbino, Italy; ^11^Department of Human Neurosciences, Sapienza University of Rome, Rome, Italy

**Keywords:** neurogenetics, Charcot-Marie-Tooth disease, multiple ligation dependent probe amplification, next-generation sequencing, diagnosis

## Abstract

Charcot-Marie-Tooth (CMT) disease is the most prevalent inherited motor sensory neuropathy, which clusters a clinically and genetically heterogeneous group of disorders with more than 90 genes associated with different phenotypes. The goal of this study is to identify the genetic features in the recruited cohort of patients, highlighting the role of rare variants in the genotype-phenotype correlation. We enrolled 67 patients and applied a diagnostic protocol including multiple ligation-dependent probe amplification for copy number variation (CNV) detection of *PMP22* locus, and next-generation sequencing (NGS) for sequencing of 47 genes known to be associated with CMT and routinely screened in medical genetics. This approach allowed the identification of 26 patients carrying a whole gene CNV of *PMP22*. In the remaining 41 patients, NGS identified the causative variants in eight patients in the genes *HSPB1*, *MFN2*, *KIF1A*, *GDAP1*, *MTMR2*, *SH3TC2*, *KIF5A*, and *MPZ* (five new vs. three previously reported variants; three sporadic vs. five familial variants). Familial segregation analysis allowed to correctly interpret two variants, initially reported as “variants of uncertain significance” but re-classified as pathological. In this cohort is reported a patient carrying a novel familial mutation in the tail domain of *KIF5A* [a protein domain previously associated with familial amyotrophic lateral sclerosis (ALS)], and a CMT patient carrying a *HSPB1* mutation, previously reported in ALS. These data indicate that combined tools for gene association in medical genetics allow dissecting unexpected phenotypes associated with previously known or unknown genotypes, thus broadening the phenotype expression produced by either pathogenic or undefined variants.

**Clinical trial registration**: ClinicalTrials.gov (NCT03084224).

## Introduction

Charcot-Marie-Tooth (CMT) disease, also known as hereditary motor and sensory neuropathy, is a common, clinically heterogeneous group of inherited peripheral neuropathies with an estimated prevalence of one in 2,500 individuals (Wiszniewski et al., 2013; Di Vincenzo et al., 2014).

CMT is clinically, neurophysiologically, and genetically heterogeneous. It is most commonly characterized by sensory loss that starts in the lower limbs and progresses slowly in a length-dependent manner. This produces progressive distal muscle atrophy, weakness, distal sensory loss, foot deformities, and depressed tendon reflexes (Shy et al., 2005; Rossor et al., 2013).

The clinical classification is based on age at onset, distribution of muscle weakness, sensory loss, walking difficulties, and foot deformities (Rossor et al., 2013). Neurophysiology allows subdividing the disease into a demyelinating (CMT1) and axonal (CMT2) forms depending on whether the median motor nerve conduction velocity (NCV) is below or above 38 m/s, respectively. A third form, intermediate CMT, has both demyelinating and axonal features and NCV between 25 and 45 m/s (Reilly and Shy, 2009; Berciano et al., 2012).

The duplication of *PMP22* is the most common cause of CMT, with a prevalence up to 40% in some populations (Reilly and Shy, 2009; Abe et al., 2011; Saporta et al., 2011; Murphy et al., 2012; Østern et al., 2013; Sivera et al., 2013). Then, approximately 100 different genes have been linked to CMT-like phenotypes which are associated with related conditions involved in axonal transport, myelin structure, and membrane metabolism that have been found in multiple unrelated families or confirmed by functional studies (Saifi et al., 2003; Saporta et al., 2011; Azzedine et al., 2012; Vallat et al., 2013; IPNMDB, 2014; OMIM, 2014; Washington University, 2014).

Therefore, the large spectrum of genetically identifiable disease alleles complicates the molecular diagnosis. Genetic heterogeneity is associated with a wide spectrum of phenotypes, complicated by the fact that mutations in the same gene cause different phenotypes (Reilly and Shy, 2009; Azzedine et al., 2012; OMIM, 2014). Furthermore, sporadic cases of CMT are not uncommon due to autosomal recessive inheritance, reduced penetrance, late-onset, small family size, and *de novo* mutations (Braathen et al., 2011; Saporta et al., 2011; Vallat et al., 2013).

In this study, we enrolled 67 patients and applied a diagnostic protocol including multiple ligation-dependent probe amplification (MLPA) for copy number variation (CNV) detection of *PMP22* locus, and next-generation sequencing (NGS) for sequencing of 47 genes known to be associated with CMT and routinely screened in medical genetics.

The goal of this study is to identify the genetic features in the recruited cohort of patients, highlighting the role of rare variants in the genotype-phenotype correlation.

## Materials and Methods

### Study Population

We collected blood or DNA samples from 67 unrelated patients with a clinical diagnosis of CMT from January 2013 to December 2019 at IRCCS Neuromed Institute (Italy). Genomic DNA was isolated from peripheral blood leukocytes according to standard procedures (QIAamp DNA Blood Mini Kit – QIAGEN).

### Clinical Data

Diagnosis of CMT includes the presence of slowly progressive neuropathy with or without family history and after exclusion of other common causes of acquired neuropathy. CMT subtype was classified as CMT if both motor and sensory nerves were similarly affected, and dHMN or HSN if the neuropathy showed exclusive or predominant involvement of motor or sensory nerves, respectively. CMT patients were further subdivided into demyelinating CMT if conduction velocity of the nondominant median or ulnar nerve was ≤38 m/s and axonal or intermediate CMT if >38 m/s.

### Literature Review

A systematic review of the literature was conducted to identify the detection rate of genetic variants and the clinical phenotype of CMT patients. Pubmed, Medline, and Embase database identified 23 cohort analysis studies consisting of Italian and European CMT patients in the period between 1997 and 2020 (Supplementary Table S1).

### Multiple Ligation-Dependent Probe Amplification

The commercially available kit P405 (MRC-Holland, Amsterdam, Netherlands) was used for the multiplex dosage. This SALSA MLPA Probemix contains 42 MLPA probes with amplification products between 130 and 445 nt: 15 probes located in the 17p12 region (*PMP22* gene), two flanking probes, seven probes in the *MPZ* gene, five probes in the *GJB1* gene, 10 reference probes detecting autosomal chromosomes, and three probes on the X-chromosome. The MLPA was performed on DNA from patients, and four normal subjects were used as internal controls.

### Next-Generation Sequencing Panel

The NGS analysis was performed using the Seq Cap EZ Choice Enrichment Kits (Hoffmann-La Roche, Basel) on an Illumina MiSeq (San Diego, CA). A full list of genes sequenced is provided in Table 1. All coding exons of the RefSeq transcripts of the genes and 15 base pairs of the flanking introns were targeted, except for *GJB1*, for which the target region is extended 860 bases upstream of the ATG start codon to include the nerve-specific promoter region. 99% of the coding exons were sequenced with a minimal read depth of 30X.

**Table 1:** Target genes included in NGS Panel.

Gene	Ref sequence	MIM
*AARS*	NM_001605.2	601065
*ALT1*	NM_005309	138200
*ARHGEF10*	NM_001308152	608236
*ATP7A*	NM_000052.6	300011
*BSCL2*	NM_001122955.3	606158
*CCT5*	NM_012073.5	610150
*DMN2*	NM_001005360.2	602378
*DYNC1H1*	NM_001376.4	600112
*EGR2*	NM_000399.3	129010
*FGD4*	NM_139241.2	611104
*FIUREG4*	NM_014845.5	609390
*GARS*	NM_002047.2	600287
*GDAP1*	NM_018972.2	606598
*GJ B1*	NM_000166.5	304040
*HSPB1*	NM_001540.3	602195
*HSPB8*	NM_014365.2	608014
*IFRD1*	NM_003640.5	603502
*IKBKAP*	NM_001197080.1	603722
*KIF1A*	NM_001244008.2	601255
*KIF1B*	NM_015074.3	605995
*KIF5A*	NM_004984.2	602821
*LITAF*	NM_004862.3	603795
*LMNA*	NM_170707.2	150330
*LRSAM1*	NM_138361.5	610933
*MED25*	NM_030973.3	610197
*MFN2*	NM_014874.3	608507
*MPZ*	NM_000530.6	159440
*MTMR2*	NM_016156.5	603557
*NDRG1*	NM_001135242.1	605262
*NEFL*	NM_006158	162280
*PMP22*	NM_000304.2	601097
*POLG*	NM_001126131	174763
*PRPS1*	NM_002764.3	311850
*PRX*	NM_181882	605725
*RAB7A*	NM_004637.5	602298
*REEP1*	NM_004637	602298
*SBF2*	NM_030962.3	607697
*SEPT9*	NM_001113491	604061
*SETX*	NM_00135152	608465
*SH3TC2*	NM_024577.3	608206
*SLC12A6*	NM_00104497.2	604878
*SMAD1*	NM_005900.3	601595
*SOD1*	NM_00045	147450
*SP110*	NM_004509	604457
*TDP1*	NM_001008744.2	607198
*SURF1*	NNM_003172.4	185620
*TGFB1*	NM_000660	190180
*TRPV4*	NM_021625.4	605427
*TTR*	NM_0209556	176300

*For each gene is reported: (i) name (GeneCards: The Human Gene Database), (ii) Ref sequence (NCBI Reference Sequence Database), and (iii) MIM (Gene/Locus MIM number)*.

GenomeUp software^1^ was used for data analysis. It provides automated annotation (Best Practices workflows of GATK v4.1 for germline variant calling), alignment of sequence reads to the reference genome GRCh37/hg19, and selection of potentially pathogenic variants. Direct evaluation of data sequence was performed by the Integrative Genomics Viewer v.2.3. Mutation re-sequencing and segregation analysis were performed by the Sanger sequencing ABI 3130xl Genetic Analyzer (Applied Biosystems).

### Data Analysis and Variant Interpretation

Variants were classified as pathogenic (class 5), likely pathogenic (class 4), and variants of uncertain significance (VoUS; class 3) according to American College of Medical Genetics Guideline for germline variant classification (Li et al., 2017). To this aim, public databases were used (VarSome https://varsome.com; GnomAD https://gnomad.broadinstitute.org). In silico analyses of variants were performed using SIFT,^2^ PolyPhen2,^3^ PROVEAN,^4^ and Mutation Assessor.^5^ The new mutations identified have been submitted in ClinVar database.^6^

## Results

### Multiple Ligation-Dependent Probe Amplification Analysis

The variation of the whole *PMP22* gene CNV was assessed through MLPA, confirming the clinical diagnosis in 26 out of 67 patients (38.8%). In detail, 20/67 (30%) were carriers of the heterozygote whole gene duplication, thus confirming the clinical diagnosis of CMT1A; 5/67 (7.4%) were carriers of the heterozygote whole gene deletion, confirming HNPP clinical diagnosis; and one was a carrier of a rare whole gene mosaic duplication of 1.5-Mb in heterozygous in *PMP22* gene (MIM 601097), considered as pathogenic (Rautenstrauss et al., 1998) and thus responsible for CMT disease 1A, autosomal dominant (MIM 118220). (Family ID 564, II:1; Figure 1A), classified as sporadic on anamnesis.

**Figure 1:**
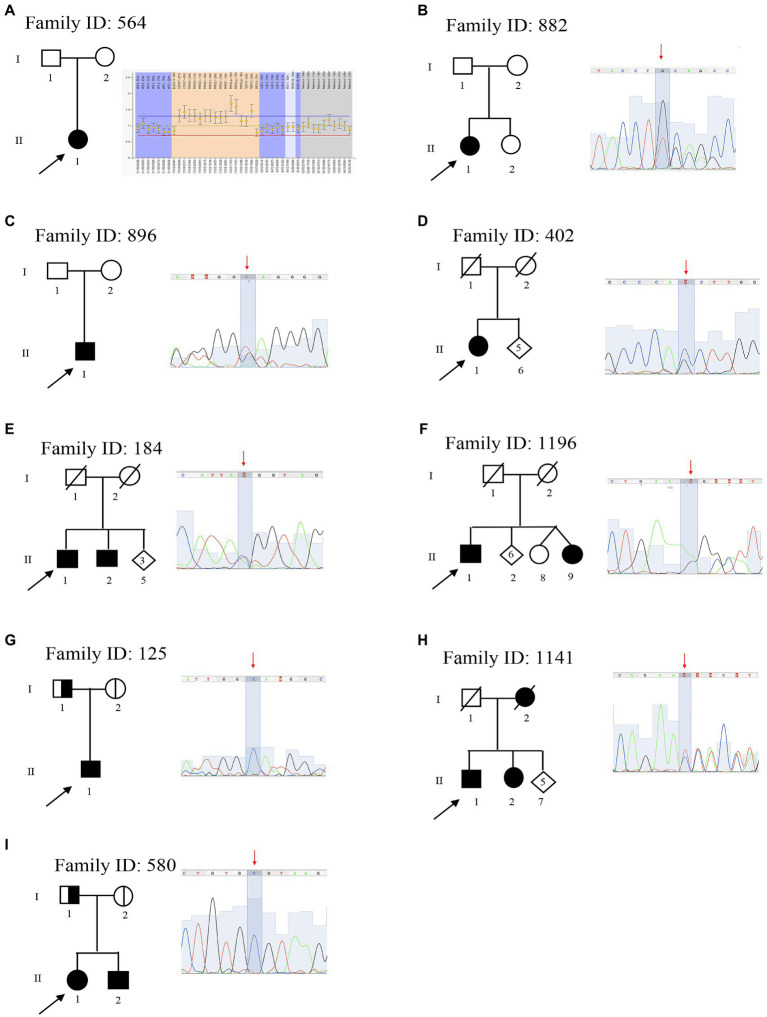
Pedigree and electropherograms of class 4 and 5 mutations identified by next-generation sequencing (NGS). **(A)** Family ID: 564, MPLA analysis and identified a mosaic duplication of 1.5-Mb (17p11.2–12); **(B)** Family ID: 882, heterozygous stop mutation in mitofusin-2 (*MFN2*) gene: NM_001127660.1:c.[2258dupT], NP_001121132.1:p.(Gln754AlafsTer9; rs773371488); **(C)** Family ID: 896, heterozygous stop mutation in *MPZ* gene: NM_000530.8: c.[306delA], NP_000521: p.(Asp104ThrfsTer14), (rs281865125); **(D)** Family ID: 402, heterozygous missense mutation in *HSPB1* gene: NM_001540.3:[c.570G > C], NP_001531.1:p.(Gln190His); **(E)** Family ID: 184, heterozygous missense mutation in *KIF1A* gene: NM_001244008.2:c.[5332C > T], NP_ p.(Arg1778Trp), (rs765668490); **(F)** Family ID: 1196, heterozygous stop mutation in *GADP1* gene: NM_018972.2: c.[140delA], NP_061845.2: p.(Lys47ArgfsTer3); **(G)** Family ID: 1251, homozygous splicing mutations in *SH3TC2* gene: NM_024577.3: c.[805 + 2T > C], (rs139052887); **(H)** Family ID: 1141, heterozygous deletion mutation in *KIF5A* gene: NM_004984.2:c.[2868_2870delTCT], NP_004975.2:p.(Leu957del), (rs575223790); and **(I)** Family ID: 580, homozygous missense mutation in *MTMR2* gene: NM_016156.5:c.[463T > C], NP_057240.3:p.(Cys155Arg).

### Next-Generation Sequencing Analysis

**Figure 2:**
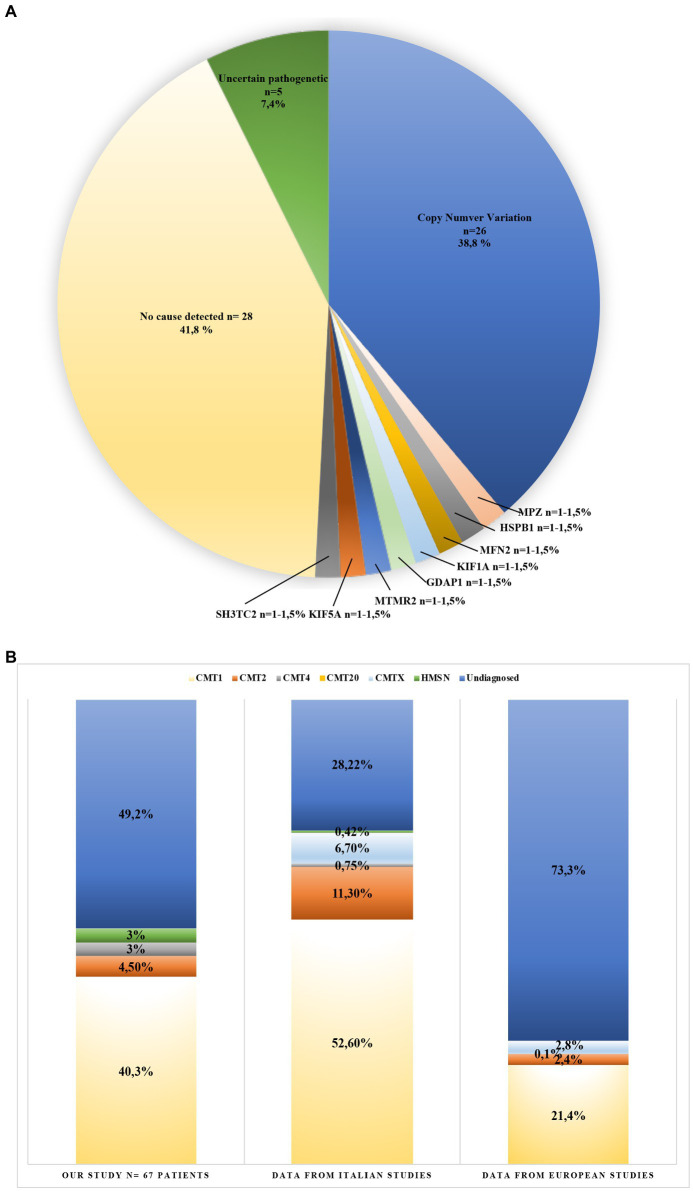
Genotype and phenotype of Charcot-Marie-Tooth (CMT) patients from this study **(A)**, and CMT patients reviewed from the literature **(B)**. **(A)** Variants identified in 67 CMT patients (blue: copy number variation 38.8%; orange: *MPZ* mutation 1.5%; gray: *HSPB1* mutation 1.5%; light yellow: *MFN2* mutation 1.5%; light blue: *KIF1A* mutation 1.5%; green: *GAPD1* mutation 1.5%; dark blue: *MTMR2* mutation 1.5%; brown: *KIF5A* mutation 1.5%; dark gray: *SH3TC2* mutation 1.5%; yellow: no cause detected 41.8%; and dark green: uncertain pathogenetic 7.4%). **(B)** This graphic compares detection yield in CMT phenotypes obtained from the literature. (light yellow: CMT1; orange: CMT2; gray: CMT4; light blue: CMTX; green: HMSN; and blue: undiagnosed). Bar 1: data obtained in this study; Bar 2: data obtained from three Italian studies; and Bar 3: data obtained from 20 European studies (see Supplementary Material).

The remaining 41 patients were tested by NGS using a target panel that considers 49 genes associated with CMT (Table 1). This approach identified the causative variants (pathogenic or likely pathogenic), in 8/41 patients (19.5%; Figure 2A). Thus, NGS improved the detection rate to 50.8% (38.8% MLPA + 12% NGS). The onset of all patients with a genetic diagnosis ranges from 7 to 57 years old, and the clinical features, consistent with phenotypes reported in OMIM database, are summarized in Table 2.

**Table 2:** Genetic and clinical data in the cohort of patients analyzed. Clinical (A) and genetic (B) features of the 15 CMT patients (16 variants) identified by NGS.

FAMID	GENE	HGVSc	HGVSp	dpSNP ID	GENOTYPE	PHENOTYPE MIM	ACMG	Clin Var	ACMG	AY	AO	Family history	CMT subtype	CV	CV subtype	Reference
882	*MFN2*	NM_001127660.1: c.[2258dupT]	NP_001121132.1: p.(Gln754AlafsTer9)	rs773371488: dupT	Heterozygous	609260	5	SCV001424047	PVS1-PM1-PM2-PP3	45	7	NO	2	>45	Normal	Our study
896	*MPZ*	NM_000530.8: c.[306delA]	NP_000521: p.(Asp104ThrfsTer14)	rs281865125 delA	Heterozygous	607791	5	SCV000928852	PVS1-PM1-PM2	45	20	NO	1 + 2	>15	Very slow	Warner et al., 1996
1196	*GDAP1*	NM_018972.2: c.[140delA]	NP_061845.2: p.(Lys47ArgfsTer3)		Heterozygous	607831	5	SCV001424519	PVS1-PM1-PM2	69	57	YES	2	>45	Normal	Our study
1251	*SH3TC2*	NM_024577.3: c.[805 + 2T > C]		rs139052887 T > C	Homozygous	601596	5	SCV001249582.3, SCV001388540.1	PVS1-PM2-PP3	45	20	YES	1	18	Slow	Piscosquito et al., 2016
564	*PMP22*	NM_153321: g.dup(17)p12			Heterozygous	118220	5	SCV001424528	PS3-PM1-PM2-PP3-PP4	44	41	NO	1	<35	Slow	Rautenstrauss et al., 1998
402	*HSPB1*	NM_001540.3: c.[570G > C]	NP_001531.1: p.(Gln190His)		Heterozygous	606595	4	SCV001424520	PM1-PM2-PP2-PP3	80	56	NO	2	38	Intermediate	Capponi et al., 2016
1141	*KIF5A*	NM_004984.2: c.[2868_2870delTCT]	NP_004975.2: p.(Leu957del)	rs575223790 del TCT	Heterozygous	604187	4	SCV001424521	PM1-PM2-PM4-PP3	60	40	YES	5	40	Intermediate	Our study
580	*MTMR2*	NM_016156.5: c.[463T > C]	NP_057240.3: p.(Cys155Arg)		Heterozygous	601382	4	SCV001424522	PM2-PM3-PP1-PP3-PP4	30	22	YES	2	<35	Intermediate	Our study
184	*KIF1A*	NM_001244008.2: c.[5332C > T]	NP_001230937: p.(Arg1778Trp)	rs765668490: C > T	Heterozygous	614213	4	SCV000952011.2	PM2-PP2-PP3-PP4	67	45	NO	5	25	Slow	Our study
721	*AGRN*	NM_198576.4: c.[5851C > T]	NP_940978.2: p.(Arg1951Cys)	rs746117937: C > T	Heterozygous	615120	3	SCV001377285.1	PM2-PP3-BP1	59	55	NO	2	>15	Very slow	Our study
721	*SCP2*	NM_002979.5: c.[886C > T]	NP_001317516.1: p.(Pro296Ser)		Heterozygous	613724	3	SCV001424523	PM2-PP3	59	55	NO	2			Our study
856	*DNM2*	NM_001005360.2: c.[890G > A]	NP_001005360.1: p.(Arg297His)	rs763894364 G > A	Heterozygous	6482	3	SCV000762731.1	PM2-PP2-PP3	67	NA	NO	1 + 2	<35	Slow	Our study
608	*MED25*	NM_030973.3: c.[949G > T]	NP_112235.2: p.(Gly317Cys)	rs1280659782 G > T	Heterozygous	605589	3	SCV001424524	PM2-PP3-BP1	53	50	NO	2	<35	Intermediate	Our study
962	*DYNC1H1*	NM_001376.4: c.[9919G > T]	NP_001367.2: p.(Val3307Leu)		Heterozygous	614228	3	SCV001424525	PM1-PM2-BP4	36	34	NO	1	20	Slow	Our study
731	*DYNC1H1*	NM_001376.4: c.[6743A > G]	NP_001367.2: p.(Glu2248Gly)		Heterozygous	614228	3	SCV001424526	PM2-PP3	63	NA		1	<35	Slow	Our study
1261	*BSCL2*	NM_001122955.3: c.[124C > T]	NP_001116427.1: p.(Arg42Cys)	rs201493373 C > T	Heterozygous	600794	3	SCV001148309.4	PP2-BS1	48	42	NO	1	>15	Very slow	Our study

*FAM ID, Family identification; HGVSc, Nomenclature human genome variation society coding DNA; HGVSp, Nomenclature human genome variation society protein; dbSNP ID, Single nucleotide polymorphism database; ACMG, American college of medical genetics guideline; AY, Years at evaluation; AO, Age at onset; CMT subtype, Demyelinating CMT=1, Axonal CMT =2, dHMN=3, HSN=4, and Possible CMT=5; CV, Conduction velocity m/s; CV subtype, CMT subtype classification for conduction velocity; PVS1, Null variant (nonsense, frameshift, canonical ±1 or 2 splice sites, initiation codon, single or multiexon deletion) in a gene where LOF is a known mechanism of disease (pathogenic and very strong); PM1, Located in a mutational hot spot and/or critical and well-established functional domain (e.g., active site of an enzyme) without benign variation (pathogenic and moderate); PM2, Absent from controls (or at extremely low frequency if recessive) in Exome Sequencing Project, 1000 Genomes Project, or Exome Aggregation Consortium (pathogenic and moderate); PM3, For recessive disorders, detected in trans with a pathogenic variant (pathogenic and moderate); PM4, Protein length changes as a result of in-frame deletions/insertions in a non-repeat region or stop-loss variants (pathogenic and moderate); PP1, Cosegregation with disease in multiple affected family members in a gene definitively known to cause the disease (pathogenic and supporting); PP2, Missense variant in a gene that has a low rate of benign missense variation and in which missense variants are a common mechanism of disease Pathogenic and supporting); PP3, Multiple lines of computational evidence support a deleterious effect on the gene or gene product (conservation, evolutionary, splicing impact, etc.; pathogenic and supporting); PP4, Patient’s phenotype or family history is highly specific for a disease with a single genetic etiology (pathogenic and supporting); BP1, Missense variant in a gene for which primarily truncating variants are known to cause disease (benign and supporting); BP4, Multiple lines of computational evidence suggest no impact on gene or gene product (conservation, evolutionary, splicing impact, etc.; benign and supporting)*.

These variants fall in eight different genes: *MFN2*, *MPZ*, *GDAP1*, *SH3TC2*, *HSPB1*, *KIF5A*, *MTMR2*, *KIF1A*, responsible for demyelinating CMT (5/64, 8%), and axonal or intermediate CMT (3/64, 4.7%; Table 2).

Of these variants, three have been previously reported as causative of CMT (Families ID 896, 125, 402; Warner et al., 1996; Capponi et al., 2016; Piscosquito et al., 2016), five are new variants (Figure 1).

In six out of eight probands, familiar members were available for segregation analysis. Of these, four variants were already classified as pathological (Families ID 882, 1141, 721, 961), while two patients were carriers of variants classified as VoUS, but reclassified as pathological through data obtained from familiar segregation (Families ID 580, 184; Figure 1). The variants identified have been classified as sporadic (3/8, 37.5%) or familiar (5/8, 62.5%).

### Causative Variants Identified as Sporadic

#### Family ID 882

The proband, a 45 years old female (II:1; Figure 1B), onset at 7 years old, presented with a moderate hyposthenia, axonal neuropathy, and normal conduction velocity (>45 m/s). She is a carrier of the new heterozygous stop mutation: NM_001127660.1:c.[2258dupT], NP_001121132.1:p.(Gln754AlafsTer9; rs773371488) in mitofusin-2 (*MFN2*; MIM 608507) considered as pathogenic and thus responsible for CMT disease 2A2A and autosomal dominant (MIM 609260). The variant was considered as sporadic since it is not present in the mother (I:2), and her father (I:1) is reported as neurological healthy.

#### Family ID 896

The proband, a 45 years old male (II:1; Figure 1C), onset at 20 years old, presented with axonal and demyelinating neuropathy with a very slow conduction velocity (>15 m/s). Molecular analysis identified the known heterozygous stop mutation: NM_000530.8: c.[306delA], NP_000521: p.(Asp104ThrfsTer14), (rs281865125) in *MPZ* (MIM 159440) considered as pathogenic (Warner et al., 1996) and thus responsible for CMT disease-dominant intermediate D (MIM 607791). The variant was considered sporadic. Family members were not available for testing, but both his mother I:2 and father I:1 were reported as neurological healthy before the age of 20 years old.

#### Family ID 402

The proband, a 76 years old female (II:1; Figure 1D), onset at 56, presented with progressive ankle instability and gait difficulties with and foot drop. She had a diagnosis of axonal neuropathy with an intermediate conduction velocity (38 m/s). Molecular analysis identified the known heterozygous missense mutation: NM_001540.3:c.[570G > C], NP_001531.1:p.(Gln190His) in *HSPB1* (MIM 602195) considered as likely pathogenic (Capponi et al., 2016) and thus responsible for CMT disease, axonal, and type 2F autosomal dominant (MIM 606595). Family members were not available for testing.

### Causative Variants Identified as Familiar

#### Family ID 184

The proband, a 67 years old male (II:1; Figure 1E), onset at 45 years old, presented with neuropathy, mild hyposthenia of the distal musculature, and hearing loss. He showed a slow conduction velocity (25 m/s) and signs of pyramidal tract dysfunction. Molecular analysis identified a new heterozygous missense mutation: NM_001244008.2:c.[5332C > T], NP_ 001230937:p.(Arg1778Trp), (rs765668490: C > T) in *KIF1A* (MIM 601255), initially considered as VoUS, but classified as likely pathogenic because of its presence in his affected brother (II:2). This variant is responsible for neuropathy, hereditary sensory, type IIC, and autosomal recessive (MIM 614213).

#### Family ID 1196

The proband, 69 years old male (II:1; Figure 1F), onset at 57 years old, presented with axonal neuropathy. He showed a normal conduction velocity. Molecular analysis identified a new heterozygous stop mutation: NM_018972.2: c.[140delA], NP_061845.2: p.(Lys47ArgfsTer3) in *GADP1* (MIM 606598) considered as pathogenic and thus responsible for CMT disease axonal, autosomal dominant, and type 2K (MIM 607831). His sister (II:9) has a similar condition but was not available for testing.

#### Family ID 125

The proband, a 45 years old male (II:1; Figure 1G), onset at 20 years old, presented with early onset demyelinating neuropathy with slow conduction velocity (18 m/s). Molecular analysis identified the known homozygous splicing mutations: NM_024577.3: c.[805 + 2T > C], (rs139052887T > C) in *SH3TC2* (MIM 608206) considered as pathogenic (Piscosquito et al., 2016) and thus responsible for CMT disease, autosomal recessive, and type 4C (MIM 601596). Both parents (I:1 and I:2) are heterozygote healthy carriers of this variant.

#### Family ID 1141

The proband a 60 years old male (II:1; Figure 1H), onset at 40 years old, presented with neuropathy and spastic paraplegia. He showed an intermediate conduction velocity (40 m/s). Molecular analysis identified a new heterozygous deletion mutation: NM_004984.2:c.[2868_2870delTCT], NP_004975.2:p.(Leu957del), (rs575223790) in *KIF5A* (MIM 602821) considered as likely pathogenic and thus responsible for spastic paraplegia 10 with neuropathy and autosomal dominant (MIM 604187). This variant is familiar since it is present in his affected sister (II:2) and absent in his healthy sister (II:4).

#### Family ID 580

The proband is a 30 years old female (II:1; Figure 1I), onset at 22, presented with axonal neuropathy and an intermediate conduction velocity (<35 m/s). Molecular analysis identified the new homozygous missense mutation: NM_016156.5:c.[463T > C], NP_057240.3:p.(Cys155Arg) in *MTMR2* (MIM 603557) initially considered as VoUS, but classified as likely pathogenic because of its presence in homozygous in her affected brother (II:2), and in heterozygous in her parents (I:1 and I:2). Thus, this variant is responsible for CMT disease, type 4B1, and autosomal recessive (MIM 601382).

### Variants Identified as VoUs

Seven VoUS were found in 6/67 patients (7%; Table 2; Figure 2A). Heterozygous variants identified in *AGRN*, *SCP2*, *DNM2*, *MED25*, *DYNC1H1*, and *BSCL2* genes associated with autosomal dominant and recessive CMT.

#### Family ID 721

The proband a 59 years old male (II:1; Figure 3A), onset at 55 years old, presented with axonal neuropathy. He showed a very slow conduction velocity (>15 m/s). He had clear psychic slowness, cognitive impairment, and floating paresthesia in the upper limbs. Molecular analysis identified two new heterozygous missense mutations: (a) in *AGRN* (MIM 103320) NM_198576.4:c.[5851C > T], NP_940978.2: p.(Arg1951Cys), (rs746117937) responsible for myasthenic syndrome, congenital, 8, with pre- and postsynaptic defects, autosomal recessive (MIM 615120), and *SCP2* (MIM 184755) NM_002979.5 c.[886C > T] and NP_001317516.1:p.(Pro296Ser) responsible for leukoencephalopathy with dystonia and motor neuropathy, autosomal recessive (MIM 613724). Both variants are not present in the healthy sister (II:2), but these data are not sufficient to consider this variant as likely pathogenic.

**Figure 3:**
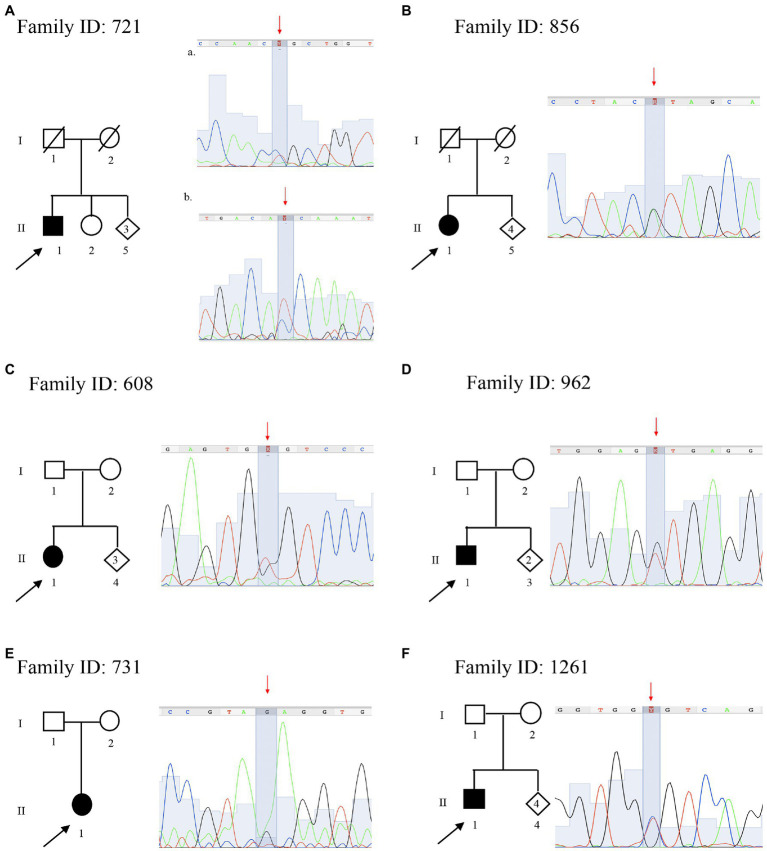
Pedigree and electropherograms of variants of uncertain significance identified by NGS. **(A)** Family ID: 721, two missense variants in (a) *AGRN* gene NM_198576.4:c.[5851C > T], NP_940978.2:p.(Arg1951Cys), (rs746117937) and (b) *SCP2* gene NM_002979.5 c.[886C > T], p.(Pro296Ser); **(B)** Family ID: 856, missense variant in *DNM2* gene NM_001005360.2:c.[890G > A], NP_001005360.1:p.(Arg297His); (rs763894364); **(C)** Family ID: 608, missense variant in *MED25* gene NM_030973.3:c.[949G > T], NP_112235.2: p.(Gly317Cys); (rs1280659782); **(D)** Family ID: 962, N missense variant in *DYNC1H1* gene NM_001376.4:c.[9919G > T], NP_001367.2:p.(Val3307Leu); **(E)** Family ID: 731, missense variant in *DYNC1H1* gene NM_001376.4:c.[6743A > G], NP_001367.2:p.(Glu2248Gly); and **(F)** Family ID: 1261, missense variant in *BSCL2* gene NM_001122955.3:c.[124C > T], NP_001116427.1:p.(Arg42Cys); (rs201493373).

#### Family ID 856

The proband, a 67 years old female (II:1; Figure 3B), presented with axonal neuropathy. She showed a slow conduction velocity (<35 m/s). Molecular analysis identified a new heterozygous missense mutation: NM_001005360.2:c.[890G > A], NP_001005360.1:p.(Arg297His; rs763894364) in *DNM2* (MIM 602378) responsible for CMT disease, axonal type 2M, and autosomal dominant (MIM 606482). Family members were not available for testing.

#### Family ID 608

The proband, a 53 years old female (II:1; Figure 3C), onset at 50, presented with axonal neuropathy. She showed an intermediate conduction velocity (<35 m/s). Molecular analysis identified a new heterozygous missense mutation: NM_030973.3;c.[949G > T], NP_112235.2: p.(Gly317Cys; rs1280659782 G > T) in *MED25* (MIM 610197) responsible for CMT disease, type 2B2, and autosomal recessive (MIM 605589). Family members were not available for testing.

#### Family ID 962

The proband, 36 years old male (II:1; Figure 3D), onset at 34, presented with demyelinating neuropathy. He showed a slow conduction velocity (20 m/s). Molecular analysis identified a new heterozygous missense mutation: NM_001376.4:c.[9919G > T], NP_001367.2:p.(Val3307Leu) in *DYNC1H1* (MIM 600112) responsible for CMT disease, type 20, and autosomal dominant (MIM 614228). Family members were not available for testing.

#### Family ID 731

The proband, 63 years old female (II:1; Figure 3E), presented with axonal neuropathy. She showed a slow conduction velocity (<35 m/s). Molecular analysis identified a new heterozygous missense mutation: NM_001376.4:c.[6743A > G], NP_001367.2:p.(Glu2248Gly) in *DYNC1H1* (MIM 600112) responsible for CMT disease, type 20, and autosomal dominant (MIM 614228). Family members were not available for testing.

#### Family ID 1261

The proband, 48 years old male (II:1; Figure 3F), onset a 42 years old, presented with demyelinating neuropathy. He showed a very slow conduction velocity (>15 m/s) and hollow foot. Molecular analysis identified a new heterozygous missense mutation: NM_001122955.3:c.[124C > T], NP_001116427.1:p.(Arg42Cys); (rs201493373) in *BSCL2* (MIM 606158), responsible for neuropathy, distal hereditary motor, and type VA (MIM 600794) autosomal dominant. Family members were not available for testing.

## Discussion

In this study, 67 patients with a clinical diagnosis of CMT were selected for molecular analysis of genes known to be related to the disease. Although the genetic of CMT is featured by a strong genetic heterogeneity, most of the patients carry 17p CNV of *PMP22*. Among this CNV, almost all cases carry the whole gene duplication of *PMP22*. The prevalence of the duplication varies among populations, ranging from 15% in Norwegian studies, up to more than 50% in Italy and Spain (Abe et al., 2011; Saporta et al., 2011; Murphy et al., 2012; Østern et al., 2013; Sivera et al., 2013; Høyer et al., 2014). In this study, only 30% of Italian patients (20/67) carry the whole gene duplication of *PMP22*, thus under-representing other Italian studies though still being in line with prevalence measured in European studies (Figure 2B).

When ruling out carriers of 17p CNV in *PMP22*, NGS identified the causative variants in 8/41 patients, which correspond only to 12% of the patients analyzed in this study (8 out of 67), being over 50% of the total of causative variants confirming the clinical diagnosis detected in the present analysis. Thus, combining various genetic tools using a target panel of 49 genes allowed to cover to a greater extent the complex clinical setting.

Several cohort studies tried to identify the diagnostic yield in CMT patients. To this aim, we conducted a systematic review of 23 cohort analysis studies consisting of Italian and European CMT patients in the period between 1997 and 2020. This data, summarized in Figure 2B and Supplementary data, identified a diagnostic yield of 71.7% in Italy (Mostacciuolo et al., 2001; Manganelli et al., 2014; Gentile et al., 2020) and 26.7% in Europe (Bort et al., 1997; Saporta et al., 2011; Murphy et al., 2012; Gess et al., 2013; Østern et al., 2013; Sivera et al., 2013; Høyer et al., 2014; Antoniadi et al., 2015; Laššuthová et al., 2016; Lupo et al., 2016; Dohrn et al., 2017; Marttila et al., 2017; Bacquet et al., 2018; Hoebeke et al., 2018; Milley et al., 2018; Nicolas et al., 2018; Lerat et al., 2019; Vaeth et al., 2019; Cortese et al., 2020; Figure 2B). Therefore, the prevalence of genetic cases of CMT provided by the present data represents an average compared with the general prevalence measured in Italy and Europe. The differences in the diagnostic rate may be explained by the differences in the specific features of the cohorts being analyzed, the number of demyelinating CMT cases enrolled, and the heterogeneous inclusion criteria considering the common causative, which were genes adopted by the previous MLPA and Sanger sequencing.

In our cohort, NGS approach identified variants in eight different genes: *MFN2*, *MPZ*, *GDAP1*, *SH3TC2*, *HSPB1*, *KIF5A*, *MTMR2*, and *KIF1A* (Table 2), five responsible for demyelinating CMT, and three for axonal or intermediate CMT. These NGS-identified variants appeared as sporadic (3/8) or familial (5/8).

A frequency of sporadic or *de novo* mutations is reported in 28–34% (Marques et al., 2005; Ando et al., 2017), although this percentage may vary and some studies report up to a half of the patients as sporadic (Ando et al., 2017). In this study, variants considered as sporadic are reported in three out of eight patients with a NGS genetic diagnosis (37.5%). This percentage, in line with other studies, may represent an overestimation since this trend cannot be verified in a few families where a full familial anamnesis is lacking. This point is crucial since it demonstrates how a careful, multifaceted genetic approach with a complete familial anamnesis and genetic segregation analysis could provide a reduction of sporadic CMT.

Among sporadic variants, the proband Family ID 564 (Figure 1A) is a carrier of a whole gene mosaic in the *PMP22* gene. This is consistent with the high instability of the *PMP22* gene which undergoes *de novo PMP22* duplications in up to 90% of sporadic CMT1 (Boerkoel et al., 2002; Marques et al., 2005).

The other probands carry sporadic variants in the gene *MFN2*, *MPZ*, and *HSPB1*. Mutations in *MFN2* cause CMT type 2A by altering mitochondrial fusion and trafficking along with the axonal microtubule system (Guerriero et al., 2020). Pathogenic variants in *MFN2* are typically inherited as autosomal dominant and are usually missense variants (Di Vincenzo et al., 2014).

The sporadic variant identified, Family ID 883 (Figure 1B), consists of a single base insertion in the coiled-coil domains producing a stop mutation p.(Gln754AlafsTer9). This could be a *de novo* variant. *De novo MFN2* mutations are regularly found in patients with a classical CMT2 phenotype (Østern et al., 2013), and some *MFN2* mutations have also been reported as *de novo* in several patients (Züchner et al., 2004; Chung et al., 2006; Verhoeven et al., 2006; Cho et al., 2007). The early onset of the proband, at 7 years old, is in line with data reporting that mutations in *MPZ*, *MFN2*, or *NEFL* are the most frequent disease causes of patients with infantile-onset CMT (Hsu et al., 2019).

Thanks to NGS studies, several data are broadening the phenotype spectrum produced by mutations in some CMT genes. For example, mutations in the N-terminal motor domain of *KIF5A* are responsible for hereditary spastic paraplegia (*SPG10* MIM 604187) and CMT type 2 (CMT2), clustered in the switch regions SWI (199–204) and SWII (232–237) necessary for microtubules interaction (Filosto et al., 2018). On the other hand, mutations in the C-terminal cargo-binding tail domain (Tail domain) are related to a specific ALS phenotype (ASL MIM 617921), with an early disease onset but a longer survival compared with typical amyotrophic lateral sclerosis (ALS; Brenner et al., 2018; Nicolas et al., 2018). Beyond these main phenotypes, other complex and overlapping phenotypes are emerging, which demonstrates that *KIF5A* mutations may be responsible for a wide range and heterogeneous disease spectrum (Schüle et al., 2008; Tessa et al., 2008). For example, in a patient, a mixed slowly progressive disease was described resembling ALS as well as HSP and axonal neuropathy. This is caused by a mutation within the terminal region of the stalk domain (Cho et al., 2007). Additionally, the patient Family ID 1141 (Figure 1H) reported in this paper represents the first case of a familial mutation, the new heterozygous deletion p.Leu957del, falling in the tail domain of *KIF5A* producing spastic paraplegia 10 with autosomal dominant neuropathy (MIM 604187).

Taken together, these results broaden the phenotypic spectrum of *KIF5A* mutations and confirm the importance of cytoskeletal defects in the pathogenesis of both ALS and CMT.

In line with this, the phenotype related to heat shock protein 27 (HSP27) is broadening. For instance, mutations in the *HSPB1* gene have been reported to cause autosomal dominant CMT with minimal sensory involvement (CMT 2F MIM 606595). Then, two independent studies reported different *HSPB1* mutations in two sporadic cases and one consanguineous family with ALS, suggesting that the disease spectrum of *HSP27* may not be limited to CMT2/dHMN (Scarlato et al., 2015; Capponi et al., 2016; Adriaenssens et al., 2017). In this study, we identified the known heterozygous missense mutation p.Gln190H in Family ID 402 (Figure 1D), which falls in the C-terminal domain involved in the control of its chaperone-like activity, responsible for CMT, axonal, and type 2F autosomal dominant (MIM 606595; Lelj-Garolla and Mauk, 2012). This variant falls in the C-terminal domain involved in the control of chaperone-like activity (Lelj-Garolla and Mauk, 2012; Capponi et al., 2016). It has been previously reported in a sporadic ALS patient with onset at 58 yo, and axonal neuropathy with an intermediate conduction velocity (38 m/s; Capponi et al., 2016). Therefore, considering this study in which the same variant has been identified in a CMT patient, the genotype-phenotype correlation is very complex. Functional characterization of different mutations located in the C-terminal domain did not show a clear correlation between the location of the mutation and associated cellular phenotype. This suggested that the genomic variant, more than its location, determines the cell pathology and phenotype, However, the present study contradicts such a conclusion which is not consistent with the present data which indicate how the same substitution has been identified in patients owing to a different disease (ALS in one case CMT in another; Capponi et al., 2016). This suggests the importance of how specific genetic backgrounds may alter the expression of the genetic variants while explaining how rare variants, like VoUS may be responsible for the disease.

In this study, seven VoUS were identified in six (7%) patients. These variants, all in heterozygous status, all fall in one of those genes associated with autosomal dominant and recessive CMT: *AGRN*, *SCP2*, *DNM2*, *MED25*, *DYNC1H1*, and *BSCL2*. This high number of VoUS is in line with the previous studies reporting a higher number of VoUS in neuropathy-associated genes, including single mutations in autosomal recessive CMT genes compared with the control population, with some patients presenting more than one VoUS in different CMT genes (Gonzaga-Jauregui et al., 2015). For instance, here, we report multiple VoUS in the proband of Family ID 721 (Figure 3A) characterized by two VoUS in *AGRN* and *SCP2*. A recent hypothesis suggests that a combinatorial effect of rare variants contributes to the disease burden in CMT and partly explains its various phenotypes. This confirms what was demonstrated by using *in vivo* experimental models in zebrafish (Cortese et al., 2020). Therefore, the interpretation of VoUS remains a key diagnostic challenge in the current NGS era.

## Conclusion

This cohort analysis demonstrates the importance of combining different molecular approaches to identify the causative variant in CMT patients. The use of NGS target panel consisting of 49 genes identified the causative variants in eight patients, improving the detection rate to 50.8%. Although this seems to represent only a small fraction of patients, the identification of rare mutations allows dissecting of unexpected phenotypes associated with previously known or unknown genotypes, thus broadening the phenotype expression produced by variants. This is the example of a patient carrying a novel familial mutation in the tail domain of *KIF5A* (a protein domain previously associated with familial ALS), and a CMT patient carrying an *HSPB1* mutation, previously reported in ALS.

In this cohort, the higher frequency of VoUS identified is in line with the previous studies, confirming that the interpretation of VoUS remains a key diagnostic challenge in the current NGS era. In line with this, in this cohort, the segregation analysis allowed to correctly interpret two variants, initially reported as VoUS, but re-classified as pathological.

To improve the detection rate in CMT patients, whole-genome sequencing (WGS) and whole-exome sequencing (WES) are strongly required. While the use of WES is now accepted for diagnostic purposes, WGS is able to both identify novel and rare variants in coding as well as noncoding regions. Therefore, these approaches allow the identification of new genes and rare variants, thus improving the genetic detection rate.

## Data Availability Statement

The datasets for this article are not publicly available due to concerns regarding participant/patient anonymity. Requests to access the datasets should be directed to the corresponding author.

## Ethics Statement

The studies involving human participants were reviewed and approved by IRCCS Neuromed Ethical Committees. The patients/participants provided their written informed consent to participate in this study. Written informed consent was obtained from the individual(s) for the publication of any potentially identifiable images or data included in this article.

## Author Contributions

CD, FB, DC, GL, CZ, SZ, AS, FF, VC, EM, EG, MM, and LS performed the recruitment and clinical evaluations of patients. RF, RC, and SS performed the genetic analyses. DC, FF, GN, MS, and SG supervised the work. RF and SG wrote the first draft of the manuscript. All authors read the manuscript, contributed to the manuscript revision, and approved the final version.

### Conflict of Interest

The authors declare that the research was conducted in the absence of any commercial or financial relationships that could be construed as a potential conflict of interest.

## Figures and Tables

**Table:** 

FAM ID	Family identification
HGVSc	Nomenclature human genome variation society coding DNA
HGVSp	Nomenclature human genome variation society protein
dbSNP ID	Single-nucleotide polymorphism database
ACMG	American College of Medical Genetics Guideline
AY	Years at evaluation
AO	Age at onset
CMT subtype	Demyelinating CMT = 1, axonal CMT = 2, dHMN = 3, HSN = 4, and possible CMT = 5
CV	Conduction velocity m/s
CV subtype	CMT subtype classification for conduction velocity
PVS1	Null variant (nonsense, frameshift, canonical ± 1 or 2 splice sites, initiation codon, single, or multiexon deletion) in a gene where LOF is a known mechanism of disease (pathogenic and very strong)
PM1	Located in a mutational hot spot and/or critical and well-established functional domain (e.g., active site of an enzyme) without benign variation (pathogenic and moderate)
PM2	Absent from controls (or at extremely low frequency if recessive) in Exome Sequencing Project, 1,000 Genomes Project, or Exome Aggregation Consortium (pathogenic and moderate)
PM3	For recessive disorders, detected in trans with a pathogenic variant (pathogenic and moderate)
PM4	Protein length changes as a result of in-frame deletions/insertions in a nonrepeat region or stop-loss variants (pathogenic and moderate)
PP1	Cosegregation with disease in multiple affected family members in a gene definitively known to cause the disease (pathogenic and supporting)
PP2	Missense variant in a gene that has a low rate of benign missense variation and in which missense variants are a common mechanism of disease (pathogenic and supporting)
PP3	Multiple lines of computational evidence support a deleterious effect on the gene or gene product (conservation, evolutionary, splicing impact, etc.; pathogenic and supporting)
PP4	Patient’s phenotype or family history is highly specific for a disease with a single genetic etiology (pathogenic and supporting)
BP1	Missense variant in a gene for which primarily truncating variants is known to cause disease (benign and supporting)
BP4	Multiple lines of computational evidence suggest no impact on gene or gene product (conservation, evolutionary, splicing impact, etc.; benign and supporting).

## Footnotes

^1^https://platform.genomeup.com

^2^http://sift.jcvi.org

^3^http://genetics.bwh.harvard.edu/pph2

^4^http://provean.jcvi.org/index.php

^5^http://mutationassessor.org

^6^https://www.ncbi.nlm.nih.gov/clinvar/
